# Changes in the Diurnal Rhythms during a 45-Day Head-Down Bed Rest

**DOI:** 10.1371/journal.pone.0047984

**Published:** 2012-10-24

**Authors:** Xiaodi Liang, Lin Zhang, Yufeng Wan, Xinyang Yu, Yiming Guo, Xiaoping Chen, Cheng Tan, Tianle Huang, Hanjie Shen, Xianyun Chen, Hongying Li, Ke Lv, Fei Sun, Shanguang Chen, Jinhu Guo

**Affiliations:** 1 Key Laboratory of Gene Engineering of the Ministry of Education, State Key Laboratory of Biocontrol, School of Life Sciences, Sun Yat-sen University, Guangzhou, China; 2 State Key Laboratory of Space Medicine Fundamentals and Application, China Astronaut Research and Training Centre, Beijing, China; 3 School of Life Sciences, University of Science and Technology of China, and Hefei National Laboratory for Physical Sciences at Microscale, Hefei, China; Morehouse School of Medicine, United States of America

## Abstract

In spaceflight human circadian rhythms and sleep patterns are likely subject to change, which consequently disturbs human physiology, cognitive abilities and performance efficiency. However, the influence of microgravity on sleep and circadian clock as well as the underlying mechanisms remain largely unknown. Placing volunteers in a prone position, whereby their heads rest at an angle of −6° below horizontal, mimics the microgravity environment in orbital flight. Such positioning is termed head-down bed rest (HDBR). In this work, we analysed the influence of a 45-day HDBR on physiological diurnal rhythms. We examined urinary electrolyte and hormone excretion, and the results show a dramatic elevation of cortisol levels during HDBR and recovery. Increased diuresis, melatonin and testosterone were observed at certain periods during HDBR. In addition, we investigated the changes in urination and defecation frequencies and found that the rhythmicity of urinary frequency during lights-off during and after HDBR was higher than control. The grouped defecation frequency data exhibits rhythmicity before and during HDBR but not after HDBR. Together, these data demonstrate that HDBR can alter a number of physiological processes associated with diurnal rhythms.

## Introduction

The simulation models for weightlessness include immersion in water, head-down bed rest (HDBR), drop tower, parabolic flight and clinostat facilities. Of these approaches HDBR is one analogue that can simulate the influence of long-term weightlessness on human physiology [1]–[2]. First adopted in the 1970s, HDBR has been proved to be useful for most gravitational biology research including cardiovascular changes, bone mass loss and muscle change in space flight [3]. In HDBR, the subjects are placed in a prone position, whereby their heads rest at an angle of 6° below horizontal. The positioning this way can lead to redistribution of the body fluid, which stimulates central volume carotid, aortic and cardiac receptors inducing an increase in diuresis and natriuresis and a decrease in plasma volume. Thus, HDBR is used to mimic the microgravity environment in orbital flight [3].

A majority of physiological, cognitive and behavioural rhythms are cooperatively controlled and synchronized by a few core circadian genes at multiple molecular levels [3]–[6]. The circadian clock governs a variety of physiological processes, and its disturbance leads to clinical and pathological conditions including sleep disorders, tumourigenesis, depression, metabolic syndrome and inflammation [3]–[4]. Moreover, the circadian clock can be entrained by a few environmental factors including light and temperature, which are called *zeitgeber*s [7]–[10].

In spaceflight, environmental factors dramatically differ from those on the Earth, e.g., light-dark cycle, illumination intensity, gravity, radiation, background noise, narrowness and isolation [11]. Apart from light and temperature, a few studies have shown that in some organisms, e.g., the filamentous fungus *Neurospora crassa* [8] and the desert beetle *Trigonoscelis gigas* [9], [10], exposure to changes in gravity may lead to an alteration of the circadian clock. Misalignment of sleep and circadian rhythm impairs the health, alertness and performance of astronauts [11]. There have been several lines of evidence suggesting that circadian rhythms might be subjective to change during space flight [12]–[16]. These facts suggest that gravity might be another factor with impact on the circadian clock. Understanding the influence of microgravity on the circadian clock is of great importance for long-duration space exploration.

The investigation of astronauts during space flight no doubt reflects the real physiological changes of circadian rhythms, but space flight also has its disadvantages. During on-orbit spaceflight, the subjects are simultaneously exposed to changes in several environmental factors than microgravity, which complicates studying the impact of each factor individually on physiology. In addition, space flights are too costly to be conducted very frequently. By far, HDBR is one of the few ground-based models for the continuous studies of physiological changes from simulated weightlessness [17].

In this work we participated in the study of a 45-day HDBR organised by the China Astronaut Research and Training Centre, which aimed to mimic actual spaceflight and to test the impact on sleep and the diurnal rhythms. We show that during this HDBR, a number of physiological variables were modified, including urinary electrolyte and hormone levels, urination and defecation frequencies, respectively.

## Subjects, Materials and Methods

### Subjects and Bioethics

This HDBR was organised by the China Astronaut Research and Training Centre. In total, eight healthy men (means±SD: age, 26.1±4.1 years; height, 171.8±3.0 cm; weight, 63.6±6.2 kg) volunteered for this study. Comprehensive physical, psychological and routine blood chemistry examinations were performed to select qualified subjects for this research, those with chronic or recent acute illness including: skeleton-muscle diseases, organic and functional diseases of psychiatry and neurology as well as sleep disorders, were excluded. The experimental conditions were well tolerated by the volunteers who completed the study and medical surveillance and service was always provided in case of emergency. No medication, smoking, alcohol, or caffeinated drinks were allowed during the study.

The meals were provided three times a day, at 7∶00, 12∶00 and 18∶00, respectively. The calorie intake data from four 72-h measurements blocks were taken: 2689.75±196.25 (R-6∼R-4 before HDBR); 2375.12±225.98 (R7∼R9 during HDBR); 2471.84±253.77 (R40–R42 during HDBR); 2675.20±319.26 (R7∼R9 after HDBR), kcal/d, respectively. The calorie intake was significantly decreased during HDBR in comparison to the control. Drinking of water was not restricted thought no significant change of the water intake amount was observed during HDBR according to the logs (data not shown).

### Bed Rest Protocol and Schedule

**Figure 1:**
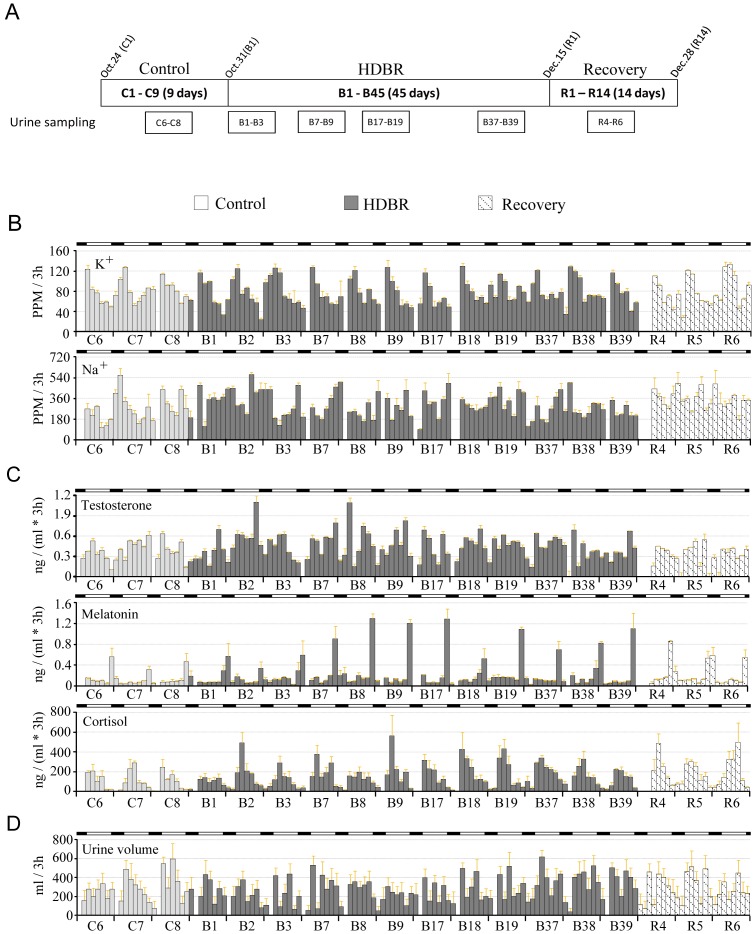
Change in urine volume, electrolyte and hormone levels. A: Diagram of the HDBR schedule. The sampling time blocks of urine are present. B - D: Change in electrolytes (B) hormone levels (C) and urine volume (D). The data were collected from measurement blocks of every 72 h (00∶00 h–11∶59 h for each day). The white bars denote day time (lights-on, 06∶00 h–21∶59 h) and the black bars denote night (lights-off, 22∶00 h–05∶59 h). The results are presented as the mean values of every 3 h ± SEM, n = 8.

The subjects were in a resting, flat, head-down position–6° from the horizontal. The entire bed rest period was composed of the 45-day HDBR, 10 days before and 10 days after the bed rest period. During all of these periods, there was intensive care monitoring (Fig. 1A).Thus the entire procedure took 65 days in total. The measurement blocks for collecting various data were also shown (Fig. 1A).

The room lighting which was turned on at 06∶00 (illumination: 300–500 lux) and turned off at 22∶00 (illumination: <10 lux) was strictly controlled. The study was performed in a quiet environment controlled at 25±2°C. The subjects attempted to relax by watching videos, listening to music, to avoid mental anguish and excessive stress.

The ambulatory period before bed rest was used as control, as previously reported [17]–[19].

### Analysis of Urinary Variables

LC–MS/MS method was employed to measure the concentrations of urinary hormone levels. The LC-MS/MS system comprised an ACQUITYUPLC system and a XEVO tandem quadrupole mass spectrometer (Eschborn, Germany), and the MassLynxv4.1 software was used to control. AQUITYUPLCBEHC_18_ 1.7 µm, 2.1×50 mm column was used for the UPLC analysis. The column temperature was 40°C and the flow was 0.6 mL/min. To obtain highest sensitivity, each sample dissolved in a 1∶1 methanol/water solution was injected separately and ionisation settings were optimised for the best fragmentation through infusion in mobile phase. Settings of a capillary voltage of 0.55 kV, cone voltage of 26–29 V, collision energy of 17–26 eV, source temperature of 150°C, desolvation temperature of 600°C, dwell time for 0.017 s, and collision cell pressure filled with argon at 4.58×10^–3^ mbar were used.

The urinary Na^+^ and K^+^ levels were analysed by an inductively coupled plasma optical emission spectrometer (Perkin-Elmer Optima 2100 ICP-OES, USA) at wavelengths of 330.237 nm for Na^+^ and 769.901 nm for K^+^. The forward power was 1.3 kW and the air flow rates for the outer, intermediate, and inner gases were 15 L/min, 0.2 L/min, and 0.8 L/min, respectively. The Na^+^ and K^+^ standards were prepared from 0.8PPM to 500PPM stock solutions. For the analysis, 200 µL of each urine sample was diluted in 2% HNO_3_ to 5 mL. A blank solution was used to set the zero emission. The emissions of all of the standards were noted and plotted against the concentrations of the standard to determine calibration line. The detection limits for the method were estimated to have a 99.5% confidence interval according to the literature [20].

Intra- and inter-assay variabilities for these assays were: 3.6% and 9.8% for melatonin, 2.2% and 8.1% for cortisol, 5.7% and 12.1% for testosterone respectively. Intra- and inter-assay variabilities were 2.7% and 11.2% for Na^+^, 4.5% and 13.6% for K^+^ respectively. The assay sensitivities were: 0.10 ng/mL for melatonin, 3.40 ng/mL for cortisol and 2.12 ng/mL for testosterone, 0.72PPM for Na^+^ (330.237 nm) and 1.31PPM for K^+^ (769.896 nm).

### Recording of Urination and Defecation

Accurate times for each voiding event of either urine or faeces were recorded for eight and six subjects, respectively. Recordings of urination were taken throughout the control, HDBR and recovery periods. Urination data were recorded for all eight subjects. The defecation frequency data were recorded for only 6 of the subjects; however, because data from the remaining two study participants were incomplete, as the participants were sent for additional studies over the course of several days during which urination, but not defecation, information was recorded. It should also be noted that the data from days 22 to 35 and days 61 to 63 were missing.

### Bioethics

The bed-rest experiment was approved by the Ethics Committee of the China Astronaut Research and Training Centre. The subjects were informed of the potential physiological and mental consequences of undergoing prolonged bed rest, and consent forms were signed. All of the subjects were required to provide written informed consent prior to their participation in the study.

### Statistical Analysis

The results are presented as the means ± SEMs. For these urinary variables, one-way repeated-measures ANOVA with factor time was calculated for each variable with a Fisher’s least significance difference (LSD) post hoc test at each time point. Lomb-Scargle periodogram analysis was utilized to assess the periodicity and significance [21]. The number of test frequencies (M) was defined as twice the number of time points in series(N), and the range of frequencies was 1/32∼1/16 in the periodicity analysis of urination, and 1/32∼1/8 in that of defecation.

## Results

### Change of Urinary Electrolyte and Hormone Profiles

We found that the urine volume displays a diurnal oscillation despite the weak rhythmicity (Fig. 1). The urine volume increased slightly but not significantly increased after initiation of HDBR (Fig. 1D). During B17–B19, the urine volume in lights-off is significantly higher than control. During B37–39, the volume increased to ∼2534 mL which is significantly higher than during the control period. After HDBR, the average urination volume dropped to a lower level comparable to that of the control (Fig. 1 and Table 1).

**Table 1:** Change of urine volume, electrolyte and hormone levels during the control, HDBR and recovery periods.

		Control (n = 8)	HDBR (n = 8)				Recovery (n = 8)
		C6–C8	B1–B3	B7–B9	B17–B19	B37–B39	R4–R6
Urine volume (mL)	Total (per day)	1916.29 (189.189)	1684.21 (131.748)	1845.79 (192.475)	1913.75 (38.216)	2565.71 (113.688)^*^	1852.03 (123.449)
	Lights-on	1619.79 (117.945)	1324.67 (154.723)	1480.50 (207.759)	1505.83 (71.541)	1994.25 (58.342)^ *^	1467.01 (74.804)
	Lights-off	296.50 (83.565)	359.54 (24.709)	365.29 (86.816)	407.92 (35.915)^ *^	571.46 (61.560)^*^	385.02 (95.601)
K^+^ (PPM)	Total (per day)	536.71 (44.035)	547.21 (31.984)	568.47 (33.089)	545.65 (19.671)	605.21 (36.358)	555.55 (97.615)
	Lights-on	405.94 (20.418)	421.46 (10.954)	423.84 (21.938)	412.65 (20.084)	409.81 (11.019)	414.53 (53.287)
	Lights-off	130.77 (24.909)	125.74 (21.968)	144.63 (31.275)	132.99 (39.472	195.40 (26.278)	141.02 (44.329)
Na^+^ (PPM)	Total (per day)	1755.48 (313.188)	2278.28 (172.256)	2093.94 (286.641)	2255.35 (298.923)	2003.48 (74.855)	2437.02 (384.648)
	Lights-on	1266.16 (138.457)	1490.54 (254.949)	1332.13 (54.823)	1585.39 (25.956)	1254.63 (44.309)	1543.73 (96.912)
	Lights-off	489.32 (174.963)	787.75 (83.673)	761.81 (244.340)	669.96 (279.613)	748.85 (106.031)	893.29 (290.730)
Cortisol (ng/mL)	Total (per day)	3287.72 (493.56)	3760.47 (343.770)	4875.77 (509.733)^*^	5480.66 (315.802)^*^	5461.12 (371.268)^*^	5296.52 (830.147)^*^
	Lights-on	3144.76 (488.281)	3434.11 (377.694)	4406.38 (523.394)	5109.82 (374.744)^*^	4652.50 (355.908)^*^	4690.44 (637.106)^*^
	Lights-off	142.96 (20.313)	326.37 (56.159)^ *^	469.39 (76.028)^ *^	370.85 (92.017)^ *^	808.627 (2.296)^*^	606.08 (21.228)^*^
Melatonin (ng/mL)	Total (per day)	1.36 (0.401)	1.67 (0.147)	1.19 (0.342)	1.85 (0.136)	2.19 (0.157)^*^	1.58 (0.273)
	Lights-on	0.72 (0.198)	0.92 (0.128)	0.58 (0.041)	0.91 (0.101)	0.95 (0.087)	0.90 (0.110)
	Lights-off	0.65 (0.206)	0.76 (0.038)	0.61 (0.367)	0.94 (0.044)	1.25 (0.243)^*^	0.68 (0.169)
Testosterone (ng/mL)	Total (per day)	2.63 (0.174)	3.14 (0.201)	3.55 (0.191)	3.25 (0.175)	3.13 (0.288)	2.14 (0.290)
	Lights-on	2.12 (0.145)	2.46 (0.214)	2.68 (0.279)	2.54 (0.251)	2.15 (0.129)	1.61 (0.099)
	Lights-off	0.51 (0.072)	0.68 (0.029)	0.87 (0.247)	0.71 (0.201)	0.98 (0.170)	0.53 (0.227)

The data are total values of urine volume and the concentration of urinary ions and hormones per day.

Data are means ± (SEM); n = 8.

One-way repeated-measures ANOVA with factor time was calculated for each variable with a Fisher’s least significance difference (LSD) post hoc test at each time point.* indicates the comparison to the control, *P*≤0.05.

Regarding hormones, melatonin and cortisol show robust diurnal rhythmicity whereas testosterone excretion barely shows diurnal rhythmicity. The cortisol excretion exhibits a diurnal pattern, whereas melatonin exhibits a nocturnal pattern (Fig. 1C). The total cortisol level and the cortisol level during lights-off of cortisol were dramatically increased throughout the HDBR and recovery periods (Fig. 1C; Table 1). The cortisol level was higher during B17–B19 and B37–B39, during lights-on (Table 1).An increase of melatonin was only found during B37–B39, in both the total and lights-off levels. For testosterone, no significant change was found throughout the bed rest despite the increase of average values (Table 1).

The fluctuation of Na^+^ levels shows poor rhythmicity, whereas the K^+^ levels exhibit reasonable diurnal rhythms (Fig. 1B). However, for both Na^+^ and K^+^, there was no overt change during the control, HDBR and recovery periods (Table 1).

### Analysis of Urination Frequency Rhythms

**Figure 2:**
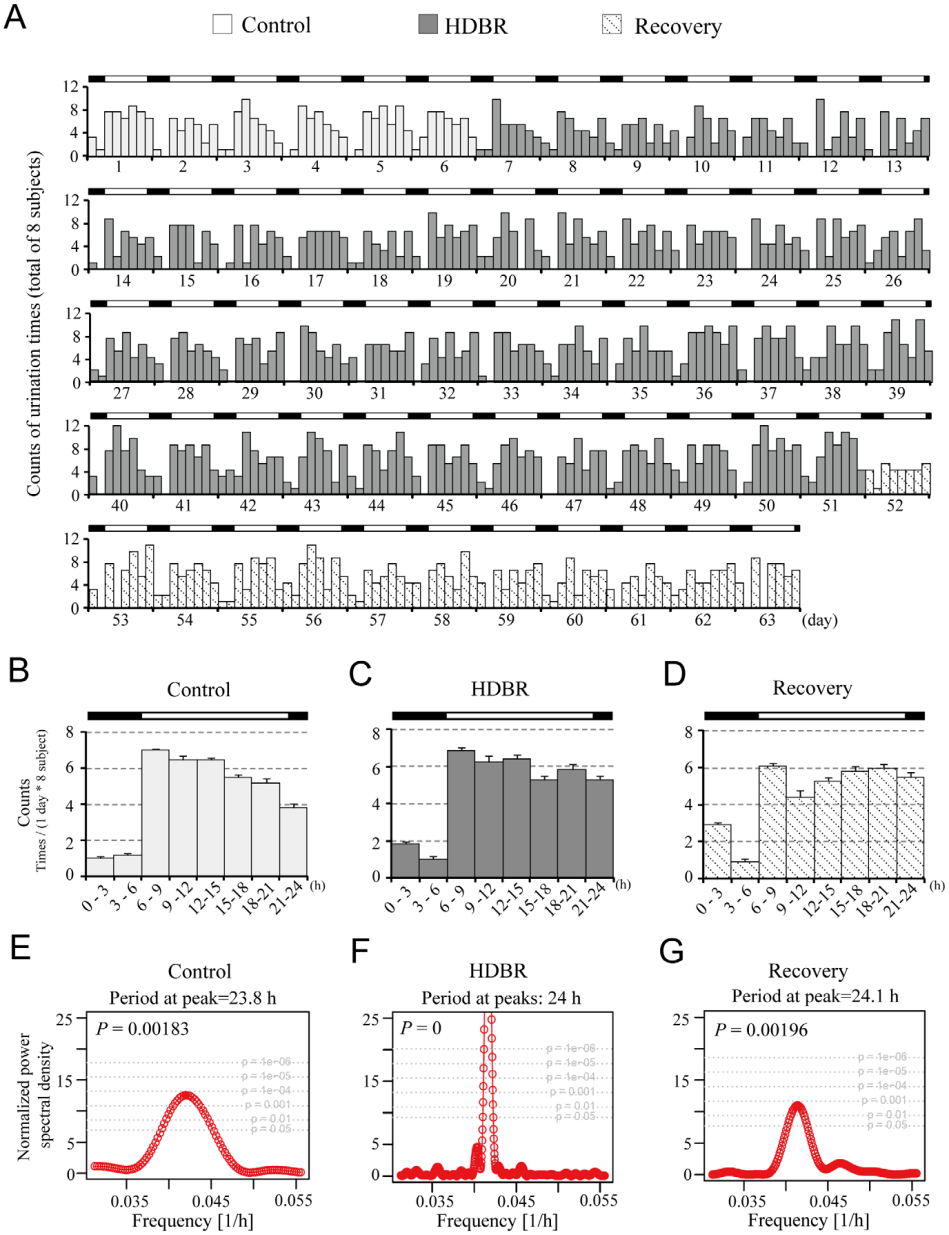
Change in urination patterns before, during and after HDBR. A: Actogram of urinary frequency. Data were taken from eight subjects, and urinary frequency was tallied and displayed every 3 h for 63 days. B – D: Average daily urinary frequency values. The data were aligned along one cycle (24 h). Data are mean ± SD. E – G: Lomb-Scargle periodgrams for the control, HDBR and recovery periods. Control is labelled in green, HDBR in orange and recovery in blue. Black bars denote light-off time (22∶00 to 06∶00), and white bars denote light-on time (06∶00 to 22∶00). *P* values indicate the statistical significance of the peaks.

Analysis of urinary frequency data in 8 Chinese men every 3 hrs shows that, during the control period, urination occurred most frequently in the daytime, peaked between 09∶00–15∶00 and occurred with the least frequency late at night and during the early morning (Fig. 2A). Under LD conditions, urinary frequency varied daily during the control period (Figs. 2A, B). In contrast, the urinary frequency patterns show less robust rhythmicity during both the HDBR and recovery periods (Figs. 2A, C, D).

To further assess the rhythmicity of urination, we conducted a Lomb-Scargle periodogram analysis and the results show peaks representing diurnal periods all around 24 h with statistic significance (Fig. 2E). These data suggest that HDBR leads to no overt influence on the diurnal rhythm of urinary frequency.

A comparison of urinary frequency during the day versus at night was also made and assessed by one-way ANOVA method. Compared with control data, urinary frequency during the lights-off period of HDBR was increased significantly, whereas the overall frequency of urination during the control, HDBR and recovery periods did not differ significantly (Table 2).

**Table 2:** Urinary frequency per day before, during and after HDBR.

	Control	HDBR	Recovery
Urinary frequency during lights-on	3.75 (0.228)	3.83 (0.102)	3.45 (0.153)
Urinary frequency during lights-off	0.75 (0.116)	1.03 (0.044)^*^	1.17 (0.083)^*^
Total urinary frequency	4.50 (0.300)	4.86 (0.121)	4.61 (0.162)

Data are means ± (SEM); n = 8.

The data were analysed by one-way analysis of variance (ANOVA) and post-hoc comparisons (Fisher’s least significant difference) methods.* *P*≤0.05.

### Analysis of Defecation Frequency Rhythms

**Figure 3:**
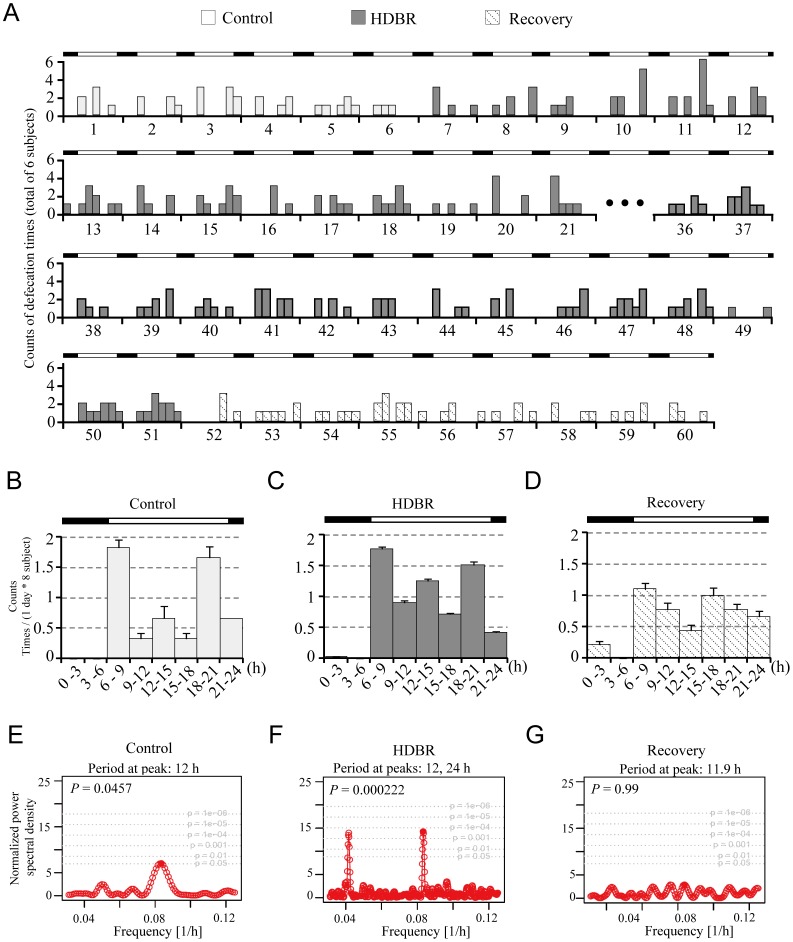
Change in defecation patterns before, during and after HDBR. A: Actogram of defecation counts. Data were taken from six subjects, and defecation counts were tallied and displayed every 3 h for 46 days. B – D: Average daily defecation counts. The data were aligned along one cycle (24 h). Data are means ± SD. E – G: Lomb-Scargle periodograms for the control, HDBR and recovery periods. Data were taken from 6 subjects, and defecation counts were tallied and displayed every 3 h for 63 days. Control is labelled in green, HDBR in orange and recovery in blue. Black bars denote lights-off time (22∶00 to 06∶00), and white bars denote lights-on time (06∶00 to 22∶00). Note that the data from days 22 to 35 and days 61–63 were incomplete and thus excluded from the analysis. *P* values indicate the statistical significance of the peaks.

Under LD conditions, the number of defecatory events for 6 of the 8 subjects was tallied every 3 hrs. The average number of events is shown in Fig. 3A. Accumulated results from all 6 subjects display weak rhythms, which, as was the case with urinary frequency, reveal that the highest occurrence of defecatory events occurs during the daytime and the lowest at night (Figs. 3B–D). During both the control and HDBR phases, the defecation patterns show roughly three peaks that occur approximately after wakening and then after lunch and again after dinner (Fig. 3B, C), which is in agreement with what Narducci et al observed [22]. However, during the recovery period, the peak incidences after morning wakening and meals were dampened (Fig. 3D), suggesting that the diurnal rhythm of defecation might be latently influenced by HDBR.

The Lomb-Scargle periodogram analysis indicated a periodic signal at peak 12 h (p<0.05) during the control analysis (Fig. 3E), which might reflect a bimodal quality of frequency [17], although a bona fide period should be close to 24 h. During HDBR, two peaks were observed at 24 h and 12 h, respectively (Fig. 3F). During the recovery period, the Lomb-Scargle periodogram analysis showed no overt of rhythmicity (*P* = 0.99) (Fig. 3G). The 12-h peaks might be caused by the bimodal profile of defecation [23], [24] and the actual period should be around 24 h. These results suggest that HDBR may lead to disturbances in the rhythm of defecation frequency.

In contrast to the diurnal distribution of urinary frequency, there were no significant differences in the number of day versus night defecatory events throughout any of the control, HDBR or recovery periods (Table 3).

**Table 3:** Defecation times per day before, during and after HDBR.

	Control	HDBR	Recovery
Defecation times during light-on	0.65 (0.060)	0.78 (0.041)	0.53 (0.091)
Defecation times during light-off	0.04 (0.042)	0.02 (0.008)	0.09 (0.020)
Total defecation count	0.69 (0.095)	0.79 (0.043)	0.63 (0.067)

Data are means ± (SEM); n = 6.

The data were analysed by one-way analysis of variance (ANOVA) and post-hoc comparisons (Fisher’s least significant difference) methods.* *P*≤0.05.

## Discussion

Microgravity affects a number of physiological functions and metabolic processes [3], [22]. Head-down bed rest (HDBR) at a −6° slope is one of the available method to simulate physiological responses to microgravity, was conducted by placing the body in a resting, flat, head-down position to –6° from the horizontal [1], [25]. Investigating sleep and the circadian clock is critical for long-duration space mission because the physiological consequences of circadian clock change in outer space remain largely unknown.

HDBR can lead to rapid diuresis and natriuresis [3]. The changes in several urinary hormones have been shown to display diurnal rhythmicity [12]. However, the urine volume change was not obvious during a 42-day bed rest [12]. To further address these questions, we tested the hormone and electrolyte levels by using UPLC-MS and ICP, respectively.

The excretion of a number of urinary electrolytes and hormones is controlled by the circadian clock [4], [26]. Bowden et al found that during bed rest, the excretion of K^+^ was unchanged but the amplitude and phase of Na^+^ excretion were altered [27]. In this work, the excretion of both Na^+^ and K^+^ was unchanged (Table 1). Millet et al showed that, during a 17-day HDBR, the rhythm in K^+^ excretion was unchanged [12], which is in agreement with our results. However, no change in Na^+^ was found in the present work whereas a decrease of Na^+^ was reported in the work of Millet et al [12]. In the LMS (Life and Microgravity Sciences) mission, cortisol excretion was significantly higher during and after spaceflight than the preflight period [28]. In accordance, we show a dramatic induction of cortisol excretion during the HDBR and recovery periods (Fig. 1; Table 1). In two space shuttle flights, the urinal cortisol excretion was reported to show a higher trend during flight though the change was not statistically significant [13]. In contrast, Millet and colleagues reported that the cortisol level was decreased in HDBR [12]. The discrepancy among the different reports might be caused by the limited number of subject, individual variance and different measurement approaches. Despite the discrepancy, these facts suggest that the profiles of hormones and electrolytes might be modified under simulated or actual microgravity conditions.

Glucocorticoid cortisol, produced by the hypothalamic-pituitary-adrenal (HPA) axis, regulates metabolism and blood sugar by mobilising stored energy for use. It has major effects on a range of physiological homeostatic mechanisms and plays an important role in stress, anxiety and depression [29]. Cortisol might suppress the excretion in testosterone [28], [30]. However, in this work we show a continuous elevation in cortisol in both HDBR and recovery while the increase of testosterone was mild. These results suggest that there is no overt negative correlation between these two hormones. High level of cortisol has been associated with osteoporosis [29], [31], [32]. Whether increase of cortisol in simulated and actual microgravity conditions accounts for weightlessness-induced bone mass loss, remains to be further investigated.

In humans, the renal function in controlling urinary excretion of water and electrolytes is controlled by an inner circadian clock [33]–[37]. Upon disruption of expression of the circadian clock gene *Per1*, the mice exhibit increased urinary sodium excretion [33]. In addition, a number of circadian clock genes and clock-controlled genes have been found in the distal convoluted tubules (DCT), the connecting tubules (CNT) and in the cortical collecting ducts (CCD) of the murine kidney [34]. With respect to defecation, most people will have a bowel movement in the morning but rarely during the night [36]–[37]. Colonic motility exhibits multimodal rhythms over a periodicity that is close to 24 h, with peak activity occurring after morning wakening and following meals [22], [38]. In ambulatory subjects, colonic pressure activity shows a wide spectrum of pressure activities around the clock [38]. In mice, measures of colonic motility and stool output exhibited circadian rhythms under both LD (Light-dark cycles) and DD (continuous dark) conditions [36]. In contrast, the rhythmicity of colonic motility was abolished in *Per1/Per2* double-knockout mice. In the mouse colon, a subset of transcripts, including neuronal nitric oxide synthase (nNOS), has been shown to be rhythmically expressed [20], [37]. These findings suggest that colonic motility is under the control of circadian regulatory patterning [36], [37].

In this work, we show that the urinary frequency during lights-off of HDBR was significantly increased during and after HDBR whereas the periodicity of urination frequency was not influenced (Table 2 and Fig. 2). For defecation, no significant change in frequency was found (Table 3). However, the impairment of defecation frequency rhythmicity was found after HDBR (Fig. 3). Melatonin and cortisol are two markers of the central circadian clock [39]. The melatonin and cortisol data exhibits robust rhythmicity throughout the HDBR experiment in the present work (Fig. 1 and Table 1), which raises the possibility that the peripheral clocks controlling urination and defecation might be modified independently. The change of food intake might also account for the alteration of defecation as there was significant decrease in calorie intake during HDBR. Whether sleep is involved in the alteration of urination and defecation remains elusive.

In conclusion, we have shown substantial evidence demonstrating the changes in a urine variables, urination and defecation, during a 45-day HDBR. The disturbance in the described parameters also suggests that a longer recovery period is crucial for a full return to baseline biological rhythms.In the future, it will be crucial to address how these physiological processes are connected.
